# Histopathologic Findings Associated With Matrix Metalloproteinases Proceeding to Recurrence of Primary Spontaneous Pneumothorax in Adolescents

**DOI:** 10.3389/fped.2021.788336

**Published:** 2021-12-01

**Authors:** Chih-Yung Chiu, Jim-Ray Chen, Shun-Ying Yin, Chia-Jung Wang, Tzu-Ping Chen, Tsan-Yu Hsieh

**Affiliations:** ^1^Department of Pediatrics and Division of Pediatric Pulmonology, Chang Gung Memorial Hospital at Linkou, College of Medicine, Chang Gung University, Taoyuan, Taiwan; ^2^Department of Pathology and Division of Pulmonary and Critical Care Medicine, Chang Gung Memorial Hospital at Keelung, College of Medicine, Chang Gung University, Taoyuan, Taiwan; ^3^Department of Surgery and Division of Thoracic and Cardiovascular Surgery, Chang Gung Memorial Hospital at Keelung, Keelung, Taiwan

**Keywords:** fibrosis, granulation tissue, macrophage accumulation, matrix metalloproteinase, primary spontaneous pneumothorax

## Abstract

**Background:** Primary spontaneous pneumothorax is potentially life-threatening, and its recurrence is always a serious problem. Pathological examination provides molecular insights into the pathophysiology of primary spontaneous pneumothorax.

**Objectives:** To investigate the association of histopathologic features of primary spontaneous pneumothorax with matrix metalloproteinase expression and their relevance to the recurrence.

**Methods:** A total of 217 tissue section slides in 172 adolescent patients with primary spontaneous pneumothorax were retrospectively reviewed from January 2001 to June 2020. All histopathologic features were recorded and pathologic findings related to ipsilateral recurrence and second surgery were analyzed. Serum levels of matrix metalloproteinases were prospectively measured in 25 primary spontaneous pneumothorax patients receiving surgery and 18 healthy controls. Their relevance to the histopathologic features of primary spontaneous pneumothorax related to its recurrence was also examined.

**Results:** The major presenting histopathologic findings of primary spontaneous pneumothorax were bleb/bulla (98%) followed by fibrosis (68%). Low prevalence of the pathologic findings of granulation tissue and macrophage accumulation were significantly associated with recurrent primary spontaneous pneumothorax, whereas fibrosis was significantly higher in patients receiving more than once surgery. Furthermore, the ratios of matrix metalloproteinase-2/tissue inhibitor of metalloproteinase-1 and matrix metalloproteinase-9/tissue inhibitor of metalloproteinase-1 were significantly higher in theses pathological findings as well as multinucleated giant cells and mesothelial cell hyperplasia in comparison with healthy controls.

**Conclusions:** Low prevalence of macrophage accumulation and granulation tissue related to the overexpression of matrix metalloproteinase-2 and−9 activities may contribute to healing impairment and primary spontaneous pneumothorax recurrence.

## Introduction

Primary spontaneous pneumothorax (PSP), characterized by the presence of air in the pleural space without apparent lung disease, affects adolescents and young adults with a significant recurrence rate (1, 2). PSP can be potentially life threatening, and the recurrence of PSP is a major long-term complication that warrants surgical intervention. Clinically, low body mass index (BMI), large-sized pneumothorax, and conservative or non-surgical treatment are risk factors for PSP recurrence (3, 4). However, a detailed understanding of the pathologic features associated with PSP and its recurrence remains lacking.

Pathological examination provides direct and powerful evidence of pathological changes and molecular insights into the pathophysiology of diseases. PSP occurs mainly from the pleural porosity of blebs or bullae. Several factors including distal airway anomaly or inflammation, apical ischemia, or abnormal connective tissue are related to the formation of blebs/bullae (5). A recent study has reported that matrix metalloproteinases (MMPs) particularly play a role in contributing to the susceptibility of the formation of bullae and areas of pleural porosity with spontaneous pneumothorax (6). However, the mechanism by which MMPs associate with the pathologic features of PSP and its recurrence remains unknown.

This study aimed to determine the histopathologic features involved in the pathogenesis of PSP and its recurrence through histological analysis of tissue slides obtained from surgery. It also aimed to determine the association of these pathologic findings with MMPs expression for PSP recurrence.

## Methods

### Study Population and Design

Adolescent patients aged 15–20 years with PSP underwent video-assisted thoracoscopic surgery (VATS) wedge resection were retrospectively enrolled from January 2001 to June 2020. PSP was defined as pneumothorax occurring in a patient without apparent underlying lung disease, trauma or inherited disorder. Clinical details including age, sex, body mass index (BMI), smoking habits, location, and recurrent side of pneumothorax were recorded and analyzed. The first VATS lung resection was considered if patients had recurrent ipsilateral pneumothorax or large size of pneumothorax with prolonged air leak over 4 days in the first episode of pneumothorax (4, 7). The second VATS surgery was performed in patients with repeated pneumothorax failure of lung expansion on the same side after the first VATS surgery. Pleurodesis was not performed routinely during VATS surgical interventions. All the histopathologic features of resected lung tissues were recorded and compared between with and without ipsilateral recurrence. Ipsilateral recurrence was defined as a further pneumothorax of the same side occurring more than 30 days apart (8) were recorded. Differences of pathologic characteristics between the first and second VATS surgery on the same side were also examined.

Adolescent PSP patients were also enrolled prospectively to investigate the pathologic findings of PSP related to matrix metalloproteinases (MMPs) serum levels. All blood samples were collected within 72 h of admission in PSP patients before the first time VATS surgery. Healthy adolescents aged <20 years from a general health check-up program without personal history of pneumothorax or other inherent pulmonary conditions were enrolled as healthy controls.

### Histological Examination

All standard hematoxylin and eosin (H&E) stained histologic sections from lung tissues of PSP patients were reviewed using light microscopy. Each tissue slide was evaluated in obvious histologic lesions and was read by the same specialist in pulmonary pathology. Pathological findings including lung tissue features (bleb/bulla, fibrosis, pathologic hyperplasia, hemorrhage, granulation tissue, and cholesterol cleft) and cell features (macrophage, giant cells, eosinophil, lymphocyte, histiocyte, and neutrophil) were recorded. Tissue pathologic images were taken and analyzed using the Olympus fluorescent microscope (Tokyo, Japan) equipped with DP71 CCD camera image capture system.

### MMPs Multiplex Assay

Serum were isolated from whole blood by centrifugation at 3,000 rpm for 10 min (Eppendorf centrifuge 5810R) and stored at −80°C until further use. MMPs measurement was performed simultaneously for the Bio-Plex Pro^TM^ Human MMP panel, 9-plex assay kit (Bio-Rad Laboratories, Hercules, CA), including MMP-1, MMP-2, MMP-3, MMP-7, MMP-8, MMP-9, MMP-10, MMP-12, and MMP-13, according to the manufacturer's instructions. Briefly, 50 ul of sample was incubated with antibody-coupled beads and biotinylated detection antibodies at room temperature. The beads were eventually re-suspended in 125 ul assay buffer and read on the Bio-Plex suspension array system. Data were analyzed using Bio-Plex Manager software version 6.0. The lower detection limit was 1.0 pg/mL with the coefficient of variation (CV) <10%.

### Enzyme-Linked Immunosorbent Assay

MMPs associated with PSP were selected for further ELISA assay. Serum levels of MMP-2, MMP-3, MMP-9, and tissue inhibitors of metalloproteinases 1 (TIMP-1) were measured using ELISA kits (DuoSet; R&D Systems; Minneapolis, USA) according to the manufacturer's instructions. The ratio of MMPs/TIMP-1 was calculated as a measure of the biological activities of MMPs.

### Statistical Analysis

The Chi-square test, χ^2^–test, or Fisher exact test was used to compare the nominal data of histopathological findings in PSP. Differences in continuous variables with non-normal distribution between PSP patients and healthy controls were tested using the Mann-Whitney test. Statistical analysis was performed using the Statistical Package for the Social Sciences (SPSS Statistics for Windows Version 20.0; Armonk, NY, USA) and graphs were drawn using GraphPad Prism Version 5.01 software (GraphPad Software Inc., San Diego, CA, USA). All statistical hypothesis tests were 2-tailed and a *P*-value < 0.05 was considered to be significant.

## Results

### Study of Pathology Slides

A total of 217 tissue section slides (101 right-sided and 116 left-sided) in 172 adolescent PSP patients with more than one pathology slide at the same time of VATS surgery were retrospectively reviewed. Among them, 19 (11%) patients had an ipsilateral recurrence after the first VATS surgery. Eighty-eight (41%) pathology slides were obtained from PSP patients with recurrence. Among them, 46 (52%) slides were right-sided recurrence, whereas 42 (48%) slides were left-sided recurrence. Pathology slides obtained from the first and second VATS on the same side were found in 192 and 25 slides, respectively (Supplementary Table 1).

### Histopathologic Features of PSP and Recurrence

**Figure 1:**
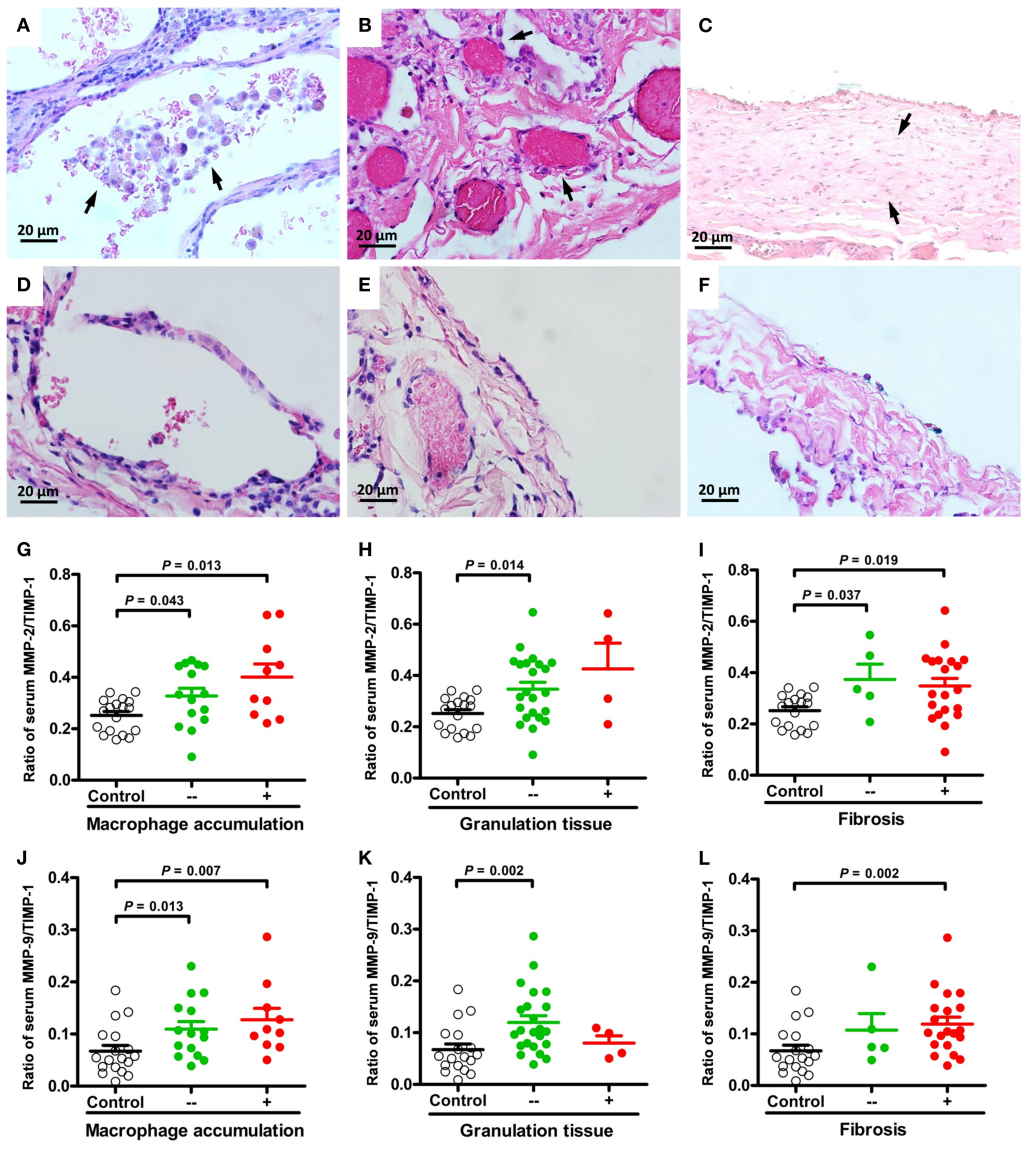
Representative micrographs (H&E, × 400) of macrophage accumulation, granulation tissue, and fibrosis in lung specimens of PSP patients. Aggregates of macrophages in the alveolar spaces [**(A)** arrows] compared to normal appearance **(D)**. Excessive proliferation of granulation tissue in the pleura [**(B)** arrows] compared to normal appearance **(E)**. Clearly delineated areas of pleural/subpleural fibrosis in non-fibrotic parenchyma [**(C)** arrows] compared to normal appearance **(F)**. Comparisons and differences of serum MMP-2/TIMP-1 and MMP-9/TIMP-1 ratios between PSP patients with and without macrophage accumulation **(G,J)**, granulation tissue **(H,K)**, fibrosis **(I,L)** and healthy controls.

The major presenting histopathologic features of PSP were bleb/bulla (98%) followed by fibrosis (68%), mesothelial cell hyperplasia (43%) and macrophage accumulation (40%). The differences in pathologic findings between adolescent PSP with and without recurrence are shown in Table 1. Compared with PSP without recurrence, there was a significantly lower prevalence of pathological findings of macrophage accumulation (Figures 1A,D) and granulation tissues (Figures 1B,E) on the right side and left side recurrent PSP, respectively (*P* < 0.05). A further logistic analysis also revealed that macrophage accumulation [odds ratio (OR) 0.434; 95% confidence interval (CI) 0.192–0.978; *P* = 0.044] and granulation tissue (OR 0.305; 95% CI 0.096–0.967; *P* = 0.044) were significantly associated with right side and left side recurrent PSP, respectively. Furthermore, fibrosis (Figures 1C,F) was significantly associated with more than once VATS (*P* < 0.01) (Table 2).

**Table 1:** Comparison and difference of pathologic characteristics between adolescent PSP with and without recurrence when having VATS.

	**Recurrence of right PSP**			**Recurrence of left PSP**		
**Pathologic characteristics**	**No (***n*** = 55)**	**Yes (***n*** = 46)**	***P***	**No (***n*** = 74)**	**Yes (***n*** = 42)**	***P***
**Tissue features**						
Bleb/bulla	55 (98%)	46 (100%)	1.000	70 (95%)	42 (100%)	0.295
Fibrosis	35 (64%)	35 (76%)	0.177	50 (68%)	28 (67%)	0.921
Mesothelial cell hyperplasia	26 (47%)	19 (41%)	0.548	29 (39%)	20 (48%)	0.377
Hemorrhage	11 (20%)	16 (35%)	0.095	22 (30%)	12 (29%)	0.895
Granulation tissue	10 (18%)	8 (17%)	0.918	19 (26%)	4 (10%)	**0.036**
Cholesterol cleft	7 (13%)	7 (15%)	0.718	16 (22%)	4 (10%)	0.097
Type II pneumocyte hyperplasia	5 (9%)	1 (2%)	0.216	7 (10%)	2 (5%)	0.485
**Cell features**						
Macrophage accumulation	30 (55%)	15 (33%)	**0.027**	29 (39%)	12 (29%)	0.250
Multinucleated giant cells	10 (18%)	10 (22%)	0.655	17 (23%)	8 (19%)	0.621
Eosinophilic infiltration	3 (6%)	3 (7%)	1.000	7 (10%)	4 (10%)	1.000
Lymphocyte aggregation	5 (9%)	6 (13%)	0.525	3 (4%)	3 (7%)	0.666
Histiocyte reaction	3 (6%)	5 (11%)	0.463	6 (8%)	3 (7%)	1.000
Neutrophil infiltration	5 (9%)	1 (2%)	0.216	4 (5%)	2 (5%)	1.000

*Data shown are number (%) of patients as appropriate. PSP, primary spontaneous pneumothorax; VATS: video-assisted thoracoscopic surgery*.

*All P-values < 0.05, which is in bold, are significant*.

**Table 2:** Comparison and difference of pathologic characteristics between adolescent PSP having the first and second VATS.

	**VATS of PSP**		
**Pathologic characteristics**	**1st (***n*** = 192)**	**2nd (***n*** = 25)**	***P***
**Tissue features**			
Bleb/bulla	187 (97%)	25 (100%)	1.000
Fibrosis	125 (65%)	23 (92%)	**0.007**
Mesothelial cell hyperplasia	79 (41%)	15 (60%)	0.074
Hemorrhage	54 (28%)	7 (28%)	0.990
Granulation tissue	40 (21%)	1 (4%)	0.054
Cholesterol cleft	31 (16%)	3 (12%)	0.774
Type II pneumocyte hyperplasia	15 (8%)	0 (0%)	0.227
**Cell features**			
Macrophage accumulation	8 (42%)	6 (24%)	0.089
Multinucleated giant cells	38 (20%)	7 (28%)	0.341
Eosinophilic infiltration	16 (8%)	1 (4%)	0.700
Lymphocyte aggregation	16 (8%)	1 (4%)	0.700
Histiocyte reaction	17 (9%)	0 (0%)	0.230
Neutrophil infiltration	11 (6%)	1 (4%)	1.000

*Data shown are number (%) of patients as appropriate. PSP, primary spontaneous pneumothorax; VATS, video-assisted thoracoscopic surgery*.

*All P-values < 0.05, which is in bold, are significant*.

### Association of MMPs With PSP Patients Receiving VATS

To evaluate the relationships between histopathologic features of PSP and MMPs, a total of forty-three subjects were prospectively enrolled, including 25 PSP patients with VATS and 18 healthy controls (Supplementary Table 2). All subjects were male and there were no significant differences in age, BMI, and cigarette smoking between healthy controls and PSP patients. Among 9 MMPs serum levels detected using Bio-Plex multiplex immunoassays; only MMP-2, MMP-3, and MMP-9 were significantly different between PSP patients with VATS and healthy controls (Supplementary Figure 1).

### Association of MMP-2, MMP-3 and MMP-9 Activity With Pathologic Findings of PSP

**Figure 2:**
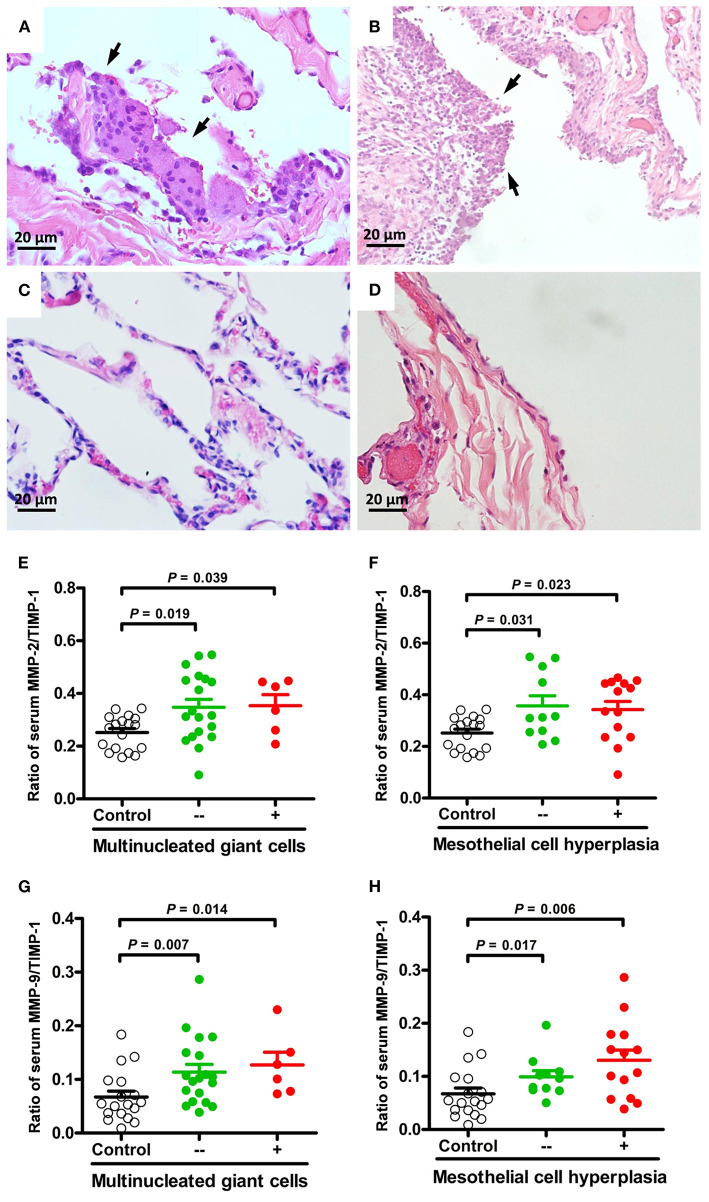
Representative micrographs (H&E, × 400) of multinucleated giant cells and mesothelial cell hyperplasia in lung specimens of PSP patients. Foci of multinucleated giant cells lining the alveolar spaces [**(A)**, arrows] compared to normal appearance **(C)**. Hyperplasia of the mesothelial cells lining the pleura [**(B)**, arrows] compared to normal appearance **(D)**. Comparisons and differences of serum MMP-2/TIMP-1 and MMP-9/TIMP-1 ratios between PSP patients with and without multinucleated giant cells **(E,G)**, mesothelial cell hyperplasia **(F,H)** and healthy controls.

The ratios of MMP-2/TIMP-1, MMP-3/TIMP-1, and MMP-9/TIMP-1 were further linked to the pathologic findings of PSP. There were no statistically significant differences in MMPs activity between histological changes of PSP. However, compared with healthy controls, MMP-2/TIMP-1 and MMP-9/TIMP-1 ratios were significantly associated with macrophage accumulation (Figures 1G,J) and granulation tissue (Figures 1H,K) related to PSP recurrence, and with fibrosis (Figures 1I,L) related to surgery. Furthermore, multinucleated giant cells (Figures 2A,C) and mesothelial cell hyperplasia (Figures 2B,D) were also significantly associated with higher ratios of MMP-2/TIMP-1 (Figures 2E,F) and MMP-9/TIMP-1 (Figures 2G,H) (*P* < 0.05), but not with MMP-3/TIMP-1 ratio.

## Discussion

PSP recurrence is a serious complication and that needs to be addressed. Histopathological examination provides unique insights into the pathogenesis and recurrence of PSP. This study has demonstrated an association between the decreased macrophage accumulation and granulation tissue formation and PSP recurrence. Overexpression of MMP-2 and MMP-9 activities appear to be associated these pathologic findings contributing to healing impairment and recurrence of PSP.

The histopathologic findings corresponding to the most common cause of pneumothorax are blebs and bullae as in this study (5). Spontaneous pneumothorax is presumed to result from the pleural porosity and air leakage of the wall of the bullae. Pathologic changes induced by cigarette smoke may lead to localized emphysema with subsequent formation of blebs or bullae (9). However, the prevalence of smoking is relatively low in this study, supporting that the formation of bullae in PSP is smoke related but not crucial (4). By contrast, a pathway-based microarray analysis revealed a breakdown of cell-extracellular matrix (ECM) interactions in the lung contributing to the formation of bullae for PSP (6).

Fibrosis, a pathological process occurring after insult to tissue injury, is characterized by the accumulation of excess ECM components with scant capillaries (10). Repeated injuries, repair, and chronic inflammation are susceptible to fibrosis in which connective tissue replaces normal parenchymal tissue leading to the formation of permanent fibrotic scar (11), which particularly explain its strong correlation with repeated surgical intervention in this study. However, an occult, repetitive lung injury with repair could be hypothesized in patients with PSP because of the high prevalence of fibrosis in this study.

Some pathologic changes are reactive and reflect responses to the injury produced by the pneumothorax. An exuberant hyperplasia of mesothelial cells in serous membranes or pneumocytes lining pulmonary alveoli is frequently related to localized atelectasis or acute lung injury (12). Multinucleated giant cells are commonly associated with blebs and persistent air leak of pleural injury (13). Chronic inflammation associated with pleural porosity contributing to air leakage can be rich in eosinophil, lymphocyte, or plasma cell aggregation as in this study. However, these cell infiltrates are inflammatory characteristics but not specific to PSP.

PSP is associated with diffuse abnormalities within the pleura, a thin tissue covered by a layer of mesothelial cells surrounding the lungs (14). Mesothelial cells possessing multiple intercellular junctions that anchor these cells link the ECM via integrins (15). Biologically, MMPs are enzymes that degrade the ECM and remodel the connective tissue. In this study, MMPs, especially MMP-2 and MMP-9, were highly expressed and strongly correlated with mesothelial cell hyperplasia in patients with PSP. These findings support the idea that an imbalance of cell–ECM interactions and MMP overexpression contribute to pleural porosity, allowing air leakage into the pleural space in PSP (6).

Granulation tissue is characterized by fibrovascular proliferation, which participates in tissue repair mechanism through the production of connective tissue protein matrix and growth of microscopic blood vessels (16). Furthermore, macrophages are the main immune cells in the granulation tissue to phagocytize damaged tissue while healing the wound. MMPs also play an important role in the formation of granulation tissue by acting on the basement membranes of arterioles. In this study, the presence of macrophage accumulation and granulation tissue was strongly associated with MMPs in patients with PSP. Furthermore, the low frequency of these pathologic findings with high risk of recurrence in patients with PSP suggests that MMPs affecting healing capacity play a crucial role in wound healing and subsequent recurrence.

MMPs are proteolytic enzymes that participate in injury and repair mechanisms by regulating immune cell influx, facilitating the migration of fibroblasts and keratinocytes, and remodeling scar tissue (17). However, uncontrolled activity of these proteases can cause healing impairments. Non-healing wounds are characterized by elevated levels and activities of MMPs, especially collagenases and gelatinases, and concomitant abnormally low levels of TIMPs (18, 19). The present study found a significantly higher activity of gelatinases (MMP-2 and MMP-9) associated with impaired granulation tissue formation, which is related to PSP recurrence. This result indicates that a reduction in MMP-2 and MMP-9 activities could promote healing and prevent PSP recurrence.

## Limitations

A major limitation of our study is its retrospective review of tissue section slides, which may show associations among variables but can rarely establish causal relationships. The relatively small sample size for the correlations between MMPs and pathologic findings of PSP may also not be representative of the entire population. Clinically, it is hindered to obtain lung tissue from adolescent healthy volunteers as controls and serum levels of MMPs and TIMPs may not reflect lung tissue levels and/or action. However, a comprehensive histological analysis of lung tissue from PSP patients with a link to the biological role of serum MMPs provides novel insights into the pathogenesis of PSP and its recurrence.

## Conclusions

A combination of histopathological examination of the lung tissues and biological activities of MMP analysis highlights the importance of an imbalance in cell-ECM interactions associated with PSP. A low prevalence of macrophage accumulation and granulation tissue is associated with increased risk of PSP recurrence. Overexpression of MMP-2 and MMP-9 activities may particularly play a role in healing impairment, leading to subsequent recurrence. However, further studies are warranted to address cause-effect relationships in studies of impact.

## Data Availability Statement

The original contributions presented in the study are included in the article/Supplementary Material, further inquiries can be directed to the corresponding author/s.

## Ethics Statement

This study was approved by the Institutional Review Board of Chang Gung Memorial Hospital (No. 103-6472C). Written informed consent to participate in this study was provided by the participants' legal guardian/next of kin. Written informed consent was obtained from the minor(s)' legal guardian/next of kin for the publication of any potentially identifiable images or data included in this article.

## Author Contributions

C-YC, T-PC, and T-YH designed and supervised the study. J-RC and S-YY provided samples and expert assistance in pathology analysis. C-JW performed experiments and analyzed the data. All authors contributed to the article and approved the submitted version.

## Funding

This work was supported by CMRPG2D0331-2 and CMRPG2B0391-2 from the Chang Gung Medical Foundation, Chang Gung University, Taiwan.

## Conflict of Interest

The authors declare that the research was conducted in the absence of any commercial or financial relationships that could be construed as a potential conflict of interest.

## Publisher's Note

All claims expressed in this article are solely those of the authors and do not necessarily represent those of their affiliated organizations, or those of the publisher, the editors and the reviewers. Any product that may be evaluated in this article, or claim that may be made by its manufacturer, is not guaranteed or endorsed by the publisher.
