# Supplementary material for: Histopathologic Findings Associated With Matrix Metalloproteinases Proceeding to Recurrence of Primary Spontaneous Pneumothorax in Adolescents
Source: Front Pediatr. 2021 Dec 1;9:788336. doi: 10.3389/fped.2021.788336 (PMC8671608; doi:10.3389/fped.2021.788336)
Supplement: Supplementary file 1 [file Data_Sheet_1.docx]

**Supplementary information**

**Histopathologic Findings Associated with Matrix Metalloproteinases Proceeding to Recurrence of Primary Spontaneous Pneumothorax in Adolescents**

Chih-Yung Chiu, Jim-Ray Chen, Shun-Ying Yin, Chia-Jung Wang, Tzu-Ping Chen & Tsan-Yu Hsieh

**Supplementary Table S1.** Baseline characteristics of 217 pathology slides in 172 adolescent PSP patients with VATS enrolled in this study.

|  | PSP patients with VATS |
| --- | --- |
| PSP patients | 172 |
| Age (yr) | 18.6 ± 2.8 |
| Sex, male | 164 (95.3%) |
| Body mass index | 18.7 ± 1.9 |
| Cigarette smoking | 41 (24.8%) |
| Pathology slides | 217 |
| Right | 101 (46.5%) |
| Left | 116 (53.5%) |
| Recurrent slides | 88 |
| Right | 46 (52.3%) |
| Left | 42 (47.7%) |
| VATS | 217 |
| The first time (1^st^) | 192 (88.5%) |
| The second time (2^nd^) | 25 (11.5%) |

Data shown are mean ± SD or number (%) of patients as appropriate. PSP, primary spontaneous pneumothorax; VATS, video-assisted thoracoscopic surgery; yr, year.

**Supplementary Table S2.** Demographic and clinical baseline characteristics of PSP patients with first time VATS and healthy controls.

|  | PSP patients with VATS (n = 25) | Healthy controls (n = 18) | *P*-value |
| --- | --- | --- | --- |
| Age (yr) | 19.0 ± 3.4 | 18.4 ± 1.4 | 0.474 |
| Sex, male | 25 (100%) | 18 (100%) | 1.000 |
| Body mass index | 19.2 ± 2.0 | 18.2 ± 2.9 | 0.355 |
| Cigarette smoking | 4 (16%) | 0 (0%) | 0.127 |
| Episodes |  |  |  |
| First attack | 10 (40%) | -- | -- |
| Non-first attack | 15 (60%) | -- | -- |
| Site |  |  |  |
| Right | 10 (40%) | -- | -- |
| Left | 15 (60%) | -- | -- |

Data shown are mean ± SD or number (%) of patients as appropriate. PSP, primary spontaneous pneumothorax; VATS, video-assisted thoracoscopic surgery; yr, year.


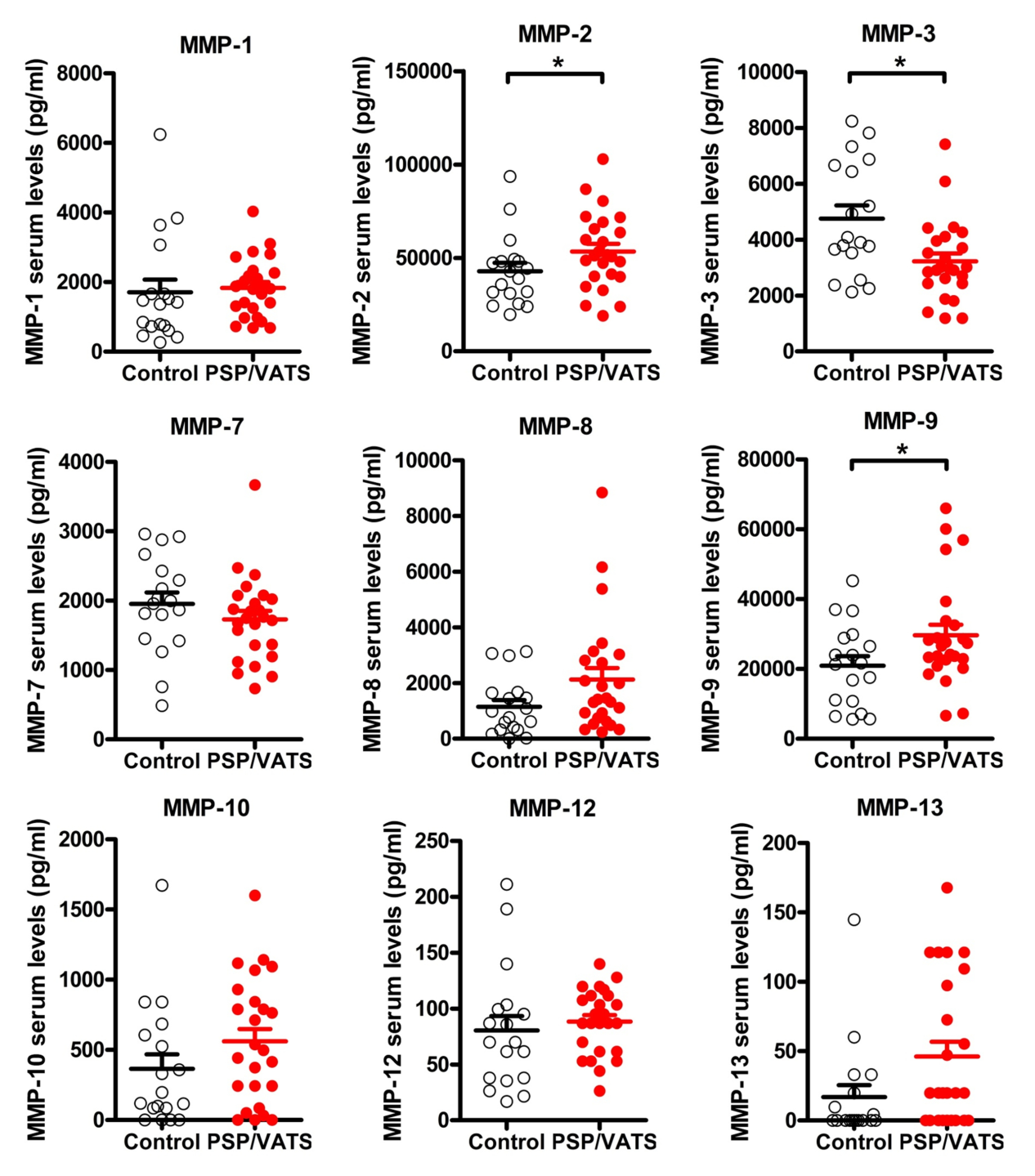


**Supplementary Figure S1.** Comparisons and differences of serum MMP-1, MMP-2, MMP-3, MMP-7, MMP-8, MMP-9, MMP-10, MMP-12, and MMP-13 levels between PSP patients receiving VATS and healthy controls. **P* < 0.05.
